# Editorial

**Published:** 1984-10

**Authors:** 


### Editorial

The attempt to broaden the basis of support for our Journal continues. As was reported in our April number overtures were made to the three Bristol Hospitals for financial help on the grounds that our journal provides an outlet for publication of work done by the staff of these hospitals. We acknowledge with gratitude the gift of £400 from the Special Trustees through the Bristol Royal Infirmary. Neither Frenchay nor Southmead Hospitals considered it 'appropriate' to support us. With the kind help of the B.M.A. approaches have been made to all doctors in the Bristol area who are not members of the Society in the hope that some of them might wish to subscribe to the Journal. Response to this has been disappointing so far. The local medical journal is an endangered species. The Medical Directory lists 33 local medical societies of which 8 style themselves 'Medico-Chirurgical'. Only 5 of these societies, including our own produce a journal. The Derby Medical Society and the Liverpool Medical Institution produce Proceedings and Transactions respectively. There is a Scottish Medical Journal produced jointly by the Edinburgh Medico-Chirurgical Society and the Royal Medico-Chirurgical Society of Glasgow and there is an Ulster Medical Journal. If the Society will accept a modest increase in its subscription rate we shall soldier on.

In this number Dr. Robin Philip contributes a thoughtful article on Undergraduate General Practice teaching in the University of Bristol. While it is accepted in principle that Bristol should have an academic department of General Practice, implementation may take time. Meanwhile Dr. Philip concludes that Bristol students are not disadvantaged. In our Correspondent's Column three contributions touch upon our relations with the Nursing profession. Dr. Whitfield describes problems which arise because decisions of a medical nature are made by senior nursing management. Dr. Mather laments the passing of hospital occasions such as nurse's prizegiving, sister's dinners, pantomimes and fancy dress dances which used to foster relationships between doctors and nurses. Lastly there is comment on the appointment of a Nurse as Manager to the South West Regional Health Authority.

